# The role of SPI1-TYROBP-FCER1G network in oncogenesis and prognosis of osteosarcoma, and its association with immune infiltration

**DOI:** 10.1186/s12885-022-09216-w

**Published:** 2022-01-25

**Authors:** Jiahua Li, Hui Shi, Zhanyuan Yuan, Zhiheng Wu, Haohao Li, Yuelong Liu, Ming Lu, Ming Lu

**Affiliations:** 1 Department of Orthopedics, Hefei BOE Hospital, Hefei, Anhui China; 2 Department of Immunology, School of Basic Medical Sciences, Anhui Medical University, No.81Mei Shan Road, Hefei, 230032 Anhui China; 3 Department of Orthopedics, the First Affiliated Hospital of Anhui Medical University, No.218 Ji Xi Road, Hefei, 230032 Anhui China

**Keywords:** Osteosarcoma, TYROBP, Immune Infiltration

## Abstract

Osteosarcoma is an aggressive malignant bone sarcoma worldwide. A causal gene network with specific functions underlying both the development and progression of OS was still unclear. Here we firstly identified the differentially expressed genes (DEGs) between control and OS samples, and then defined the hub genes and top clusters in the protein–protein interaction (PPI) network of these DEGs. By focusing on the hub gene *TYROBP* in the top 1 cluster, a conserved *TYROBP* co-expression network was identified. Then the effect of the network on OS overall survival was analyzed. Gene Ontology (GO), Kyoto Encyclopedia of Genes and Genomes (KEGG) enrichment analyses and Gene Set Enrichment Analysis (GSEA) were used to explore the functions of the network. XCell platform and ssGSEA algorithm were conducted to estimate the status of immune infiltration. ChEA3 platform, GSEA enrichment analysis, and Drug Pair Seeker (DPS) were used to predict the key transcription factor and its upstream signal. We identified the downregulated SPI1-TYROBP-FCER1G network in OS, which were significantly enriched in immune-related functions. We also defined a two-gene signature (*SPI1*/*FCER1G*) that can predict poorer OS overall survival and the attenuated immune infiltration when downregulated. The SPI1-TYROBP-FCER1G network were potentially initiated by transcription factor SPI1 and would lead to the upregulated CD86, MHC-II, CCL4/CXCL10/CX3CL1 and hence increased immune infiltrations. With this study, we could better explore the mechanism of OS oncogenesis and metastasis for developing new therapies.

**Supplementary Information:**

The online version contains supplementary material available at 10.1186/s12885-022-09216-w.

## Introduction

Osteosarcoma (OS) is the most common primary malignant bone sarcoma worldwide, particularly in children and adolescents [1, 2]. OS is a highly invasive tumor with 20% of patients being diagnosed at metastatic stage and 50% of patients with lung metastases at late stage. OS was due to a dysfunctional osteoblastic differentiation from the primitive mesenchymal bone-forming cells [3]. Now the treatment for OS has reached a plateau to the patients with metastases, the 5-year survival rate of which is under 30% [4]. Therefore, identifying the key gene network and their molecular mechanisms underlying both the pathogenesis and progression of OS is necessary and urgent.

Some studies have been published, which focused on the role of single oncogene in OS, such as p53 (arresting cell cycle and promoting apoptosis) [5], SOX2 (enhancing cell stemness and migration) [6], MALAT1 (promoting proliferation and metastasis) [7], IGF-2 (affecting osteoblast differentiation) [8], cyclin E1 (inhibiting proliferation and increasing chemotherapeutics sensitivity) [9], and PTH (affecting migration) [10]. In recent years, some studies identified a cluster of hub genes as OS biomarkers via differentially expressed genes (DEGs) analysis followed by protein–protein interaction (PPI) network analysis or weighted gene co-expression network analysis (WGCNA). In this kind of analyses, multiple clusters of hub genes have been identified as OS characteristic biomarkers or metastatic biomarkers involving functions such as focal adhesion, type I interferon signaling and cell–cell adhesion [11–15]. However, a conserved gene network involved in both OS oncogenesis and OS prognosis have not been fully analyzed. Furthermore, the common gene network and functions across cancer types have not been fully discussed.

Here we integrated DEG results from GSE33382, GSE12865, GSE16088 and GSE14359 datasets and analyzed the survival data from TARGET-OS and GSE21257 datasets to identify the immune-infiltration associated gene network (i.e., SPI1-TYROBP-FCER1G network) underlying both OS oncogenesis and prognosis. Firstly, we identified the DEGs and defined the hub genes and top clusters in the PPI network of those DEGs. Secondly, we did univariate Cox regression analysis to identify the 9 prognostic genes in the five top clusters. To furtherly explore the function of TYROBP-centered top 1 cluster, we did an analysis across cancer types to identify a more conserved and extensive TYROBP co-expression network, which was found to be significantly associated with leukocyte proliferation and T cell activation later. By PPI analysis and least absolute shrinkage and selection operator (LASSO) Cox regression analysis on the TYROBP co-expression network, the two-gene signature of FCER1G and SPI1 were identified as a novel prognosis biomarker. Furthermore, the transcriptional levels of FCER1G and SPI1 can also distinguish the OS patients into two clusters with different prognosis and immune cell infiltrations, suggesting a potential treatment target for OS and other TYROBP-associated cancers.

## Materials and methods

### Data resource and processing

Four microarray datasets of OS samples were collected from GEO database, including GSE33382, GSE12865, GSE16088 and GSE14359. The selection criteria included: 1. including both normal control and osteosarcoma tissues; 2. from Homo sapiens; 3. more than 10 samples. The platform annotation documents were also downloaded from GEO and annotated for microarray probes by “merge” R command.

Both expressional data and survival data from TARGET-OS and GSE21257 datasets were downloaded for analyzing the effect of gene expression on prognosis (1. including both osteosarcoma tissue and survival data; 2. from Homo sapiens; 3. more than 20 samples).

TCGA Pan-Cancer TPM data (TOIL workflow processed [16]) were obtained from UCSC Xena Browser (https://xenabrowser.net/datapages/). The correlations between the expressional levels of *TYROBP* and other genes were assessed using Spearman correlation analysis (*R* > 0.6 and *p* < 0.05 as significance). GEPIA2 platform was used to analyze the correlation between *SPI1*, *TYROBP* and *FCER1G* across multiple cancer types [17].

ENCODE PU.1 ChIP-seq peaks data from multiple cell lines (i.e., GM12878, GM12891, K562, HL-60) were downloaded [18] and visualized by software IGV [19]. RNA-seq and ChIP-seq data from THP-1 cell line were downloaded from GSE69284 and GSE89178, the peaks of which were called by HOMER [20]. ChIP-X Enrichment Analysis 3 (ChEA3) platform was used for transcription factor (TF) prediction by transcription factor enrichment analysis that ranks TFs associated with user-submitted gene sets [21].

### Analysis of differentially expressed genes

GEO2R online tool was used to systematically measure the DEGs in GSE33382, GSE12865 and GSE14359 (between control and OS). ‘GEOquery’ R package and ‘limma’ R package were used to measure the DEGs in GSE16088 (between control and OS) [22, 23]. Significant DEGs were selected by Benjamini & Hochberg (BH, False discovery rate) adjusted *p* < 0.05 and |log2 fold change (FC)|> 0.58. Then the overlapped results were visualized by Venn diagram via “ggplot2” R package [24].

### Functional enrichment analysis

Gene Ontology Analysis on Gene Clusters Gene ontology (GO) (including biological process (BP), molecular functions (MF), and cellular components (CC) terms) [25] and Kyoto Encyclopedia of Genes and Genomes (KEGG) pathways [26] are used to annotate the functions of genes via “clusterProfiler” R package [27] and virtualized by “ggplot2” R package.

Gene set enrichment analysis (GSEA) was performed by pre-ranking genes based on R value of TYROBP correlation level. We subsequently run the GSEA preranked analysis with the c2.cp.reactome.v7.2.symbols.gmt (reactome), h.all.v7.2.symbols.gmt (signaling pathway), and c3.all.v7.2.symbols.gmt (transcriptional factor motif) gene set from MsigDB [28] via “clusterProfiler” R package.

### Protein–protein interaction network

The Search Tool for the Retrieval of Interacting Genes (STRING) database is an online tool that is used to develop protein–protein interaction (PPI) networks [29]. The network data of proteins from DEGs were downloaded from STRING to visualize the protein interaction network in the Cytoscape software [30]. In the network of DEGs, the 5 top clusters (highly interconnected regions) were identified by MCODE plugin in Cytoscape, which can find clusters (i.e., the highly interconnected regions) by vertex weighting and isolating the dense regions in a network [31]. The degree of gene connection was analyzed by Cytoscape and reflected by its area. In the TYROBP network, we identified the ranked hub genes by cytoHubba plugin (with Maximal Clique Centrality (MCC) algorithm) [32] in Cytoscape.

### Survival analyses

Survival analyses were performed using univariate Cox regression and LASSO regression via “survival” [33] and “glmnet” [34] R package. For LASSO regression, the final formula of the risk signature was given as risk score = expression level of Gene1*β1 + expression level of Gene2*β2 + … + expression level of Genen* βn, in which β represents the regression coefficient of each variable. The receiver operating characteristic (ROC) curves for the imaging markers were constructed for overall survival, and the areas under the ROC curves (AUC) were estimated empirically with the trapezoid rule with “pROC” [35] and “ggplot2” R package.

### Immune infiltration analysis

Platform xCell (xCell signature) [36] and single-sample gene set enrichment analysis (ssGSEA, based on immune signature by “gsva” R package [37]) were used to reckon the proportion and score of immune cell infiltration in OS tumor environment, respectively. We filtered out the cell types with proportion less than 0.01 in xCell results. Boxplots of proportions and normalized scores were performed with “ggplot2” R package. Wilcox test was performed for the comparison between groups. ssGSEA, as an extension of GSEA, can calculate separate enrichment scores of immune cells for the pairing of OS samples and the gene set of immune cell signatures. XCell can calculate separate proportions of immune cells in microenvironment based on gene signatures for 64 cell types.

## Results

### The DEGs from multiple OS datasets and the immune-related function of downregulated genes

**Fig. 1:**
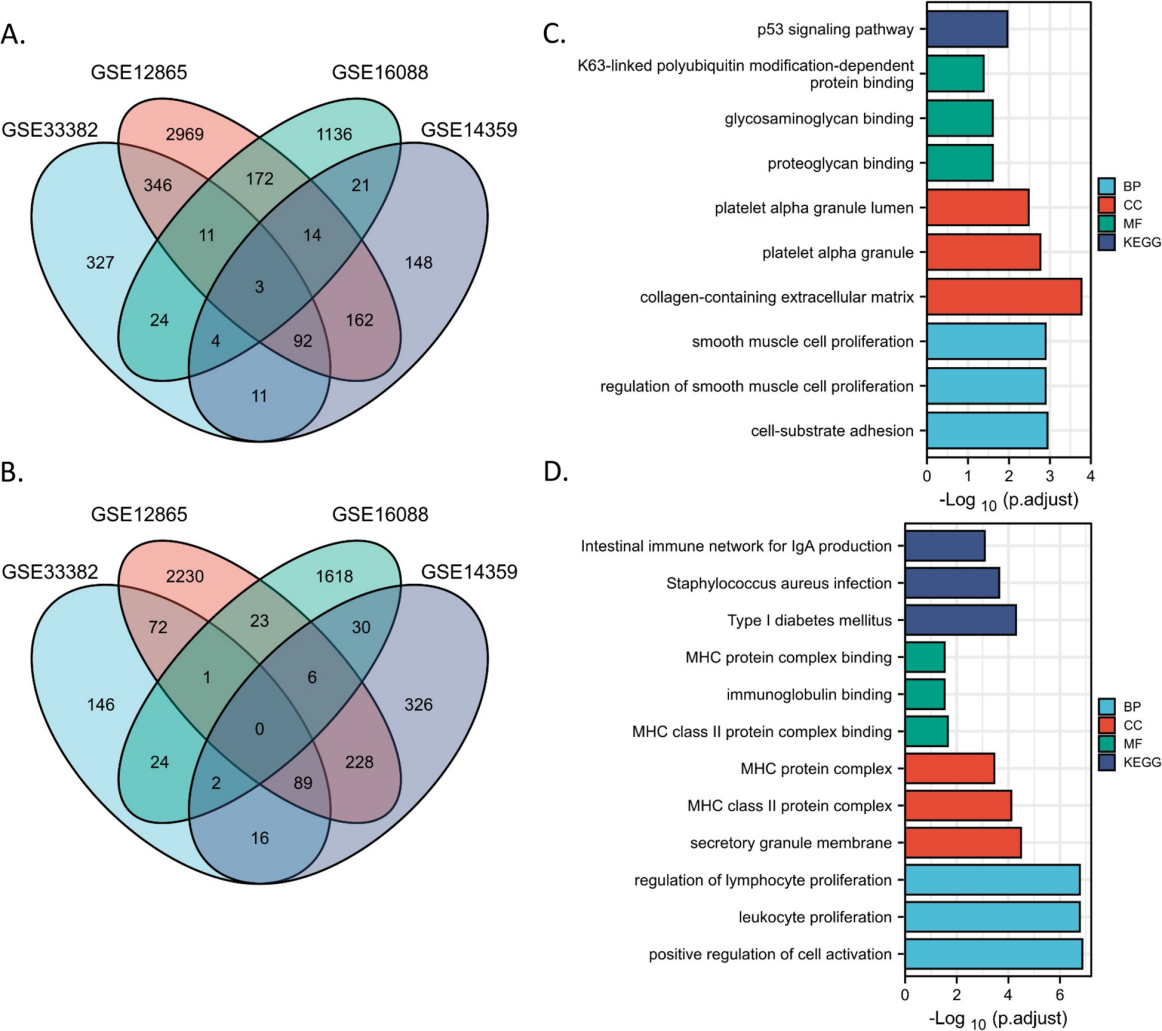
Differentially expressed genes (DEGs) were identified in four OS datasets and functional enrichment analysis on them. **A-B** The Venn diagram representing DEGs numbers in each dataset. **C-D** The top three KEGG pathways and GO functions (BP, CC, MF) assembled by upregulated (**C**) and downregulated (**D**) genes

Four of GEO datasets with both normal control and osteosarcoma tissues were included in the study. It’s notable that there were a few of differences between these datasets, reflecting the complementarity between them. Firstly, in GSE12865 and GSE14359, the control samples were normal primary osteoblasts, while in GSE33382 the control samples were osteoblasts derived from osteogenic differentiated bone-marrow-derived mesenchymal stem cells. In GSE16088, the control samples were from an unspecific osteoblast cell line H-012706. Furthermore, although OS samples were all OS tissues not cell line in the four datasets, in GSE33382 the OS samples were from high-grade OS pre-chemotherapy biopsy. In GSE12865, OS samples were all from pediatric OS patients. In GSE14359, OS group included a part of lung metastasis OS tissues. For a comprehensive analysis, the DEGs ( |log_2_(Fold Change)|> 0.58 and adjusted *p* < 0.05) were identified in 4 OS datasets independently (Supplementary Fig. 1A). The overlapped DEGs in >  = 3 datasets were considered as credible DEGs for the next functional enrichment analysis (Fig. 1A-B). In the end, we found 124 of upregulated genes (Fig. 1A) and 98 of downregulated genes (Fig. 1B).

Among them, the 124 upregulated genes were enriched in annotations such as collagen-containing extracellular matrix, smooth muscle cell proliferation, cell-substrate adhesion and p53 signaling pathway (Fig. 1C), while the 98 downregulated genes were enriched in immune-related functions including MHC class II protein complex/complex binding, lymphocyte/leukocyte proliferation, and so on, indicating a significant loss of MHC class II protein complex and immune cell activation ability in OS samples compared with that in control samples (Fig. 1D).

### The hub genes and top clusters in PPI network of DEGs, and the immune-related function of top 1 cluster genes

**Fig. 2:**
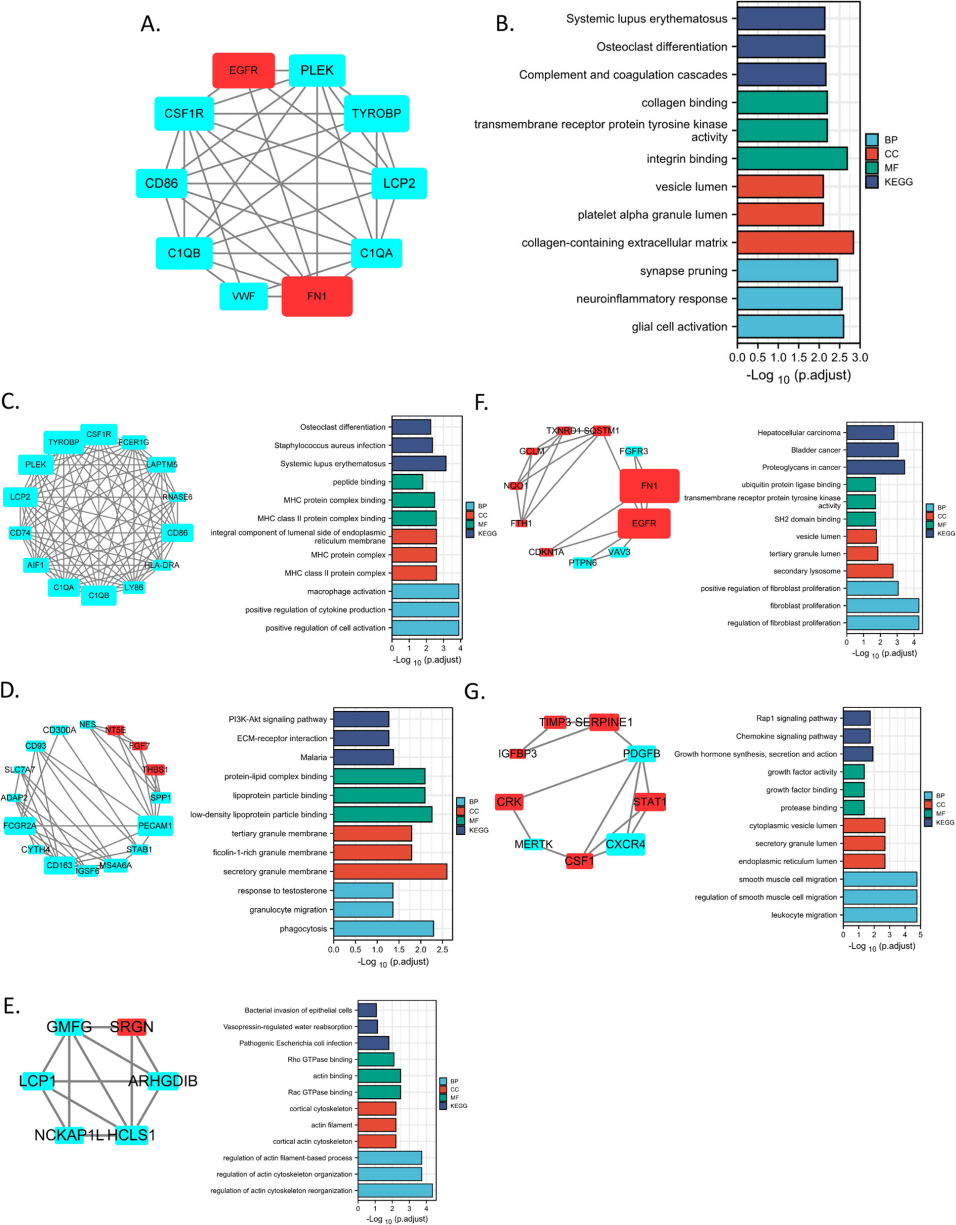
The top 10 hub genes in PPI network of DEGs and the top 5 cluster genes in DEGs network. **A** The top 10 hub genes in PPI network of DEGs. The area represented the degree of each node the red node represented upregulated genes, while the blue node represented the downregulated genes. **B** The top 3 KEGG and GO annotations enriched by the total DEGs. **C-G** The PPI network of top 5 clusters

To define the hub genes and functional gene clusters in DEGs, we put upregulated and downregulated genes together to construct the PPI network by Cytoscape based on STRING database. According to the connection degrees of DEGs, we found the hub genes with the top 10 degrees (reflected by their areas in Fig. 2A) in the network (Supplementary Fig. 1B) including *FN1*, *TYROBP*, *EGFR*, *CSF1R*,*C1QB*, *PLEK*, *LCP2*, *C1QA*, *CD86* and *VWF*, which were enriched in functions including osteoclast differentiation/integrin binding/collagen-containing extracellular matrix/glial cell activation (Fig. 2B), and potentially initiated the whole PPI network as the hub genes.

Furthermore, we defined the top 5 clusters by MCODE plugin in Cytoscape and analyzed their functions respectively (Fig. 2C-G). Notably, genes from top 1 cluster were all downregulated in OS and enriched in immune-related functions such as positive regulation of immune cell activation/cytokine production, and MHCII class II protein complex (Fig. 2C). Top 2 cluster genes were about the functions of lipoprotein particle binding, granule membrane and phagocytosis (Fig. 2D). Top 3 cluster genes were enriched in functions of actin cytoskeleton reorganization (Fig. 2E). Top 4 cluster genes were mainly associated with fibroblast proliferation (Fig. 2F). Top 5 cluster genes were most significantly associated with cell migration including smooth muscle cell migration and leukocyte migration (Fig. 2G).

### Five genes from top 1 cluster can also predict patient prognosis

**Fig. 3:**
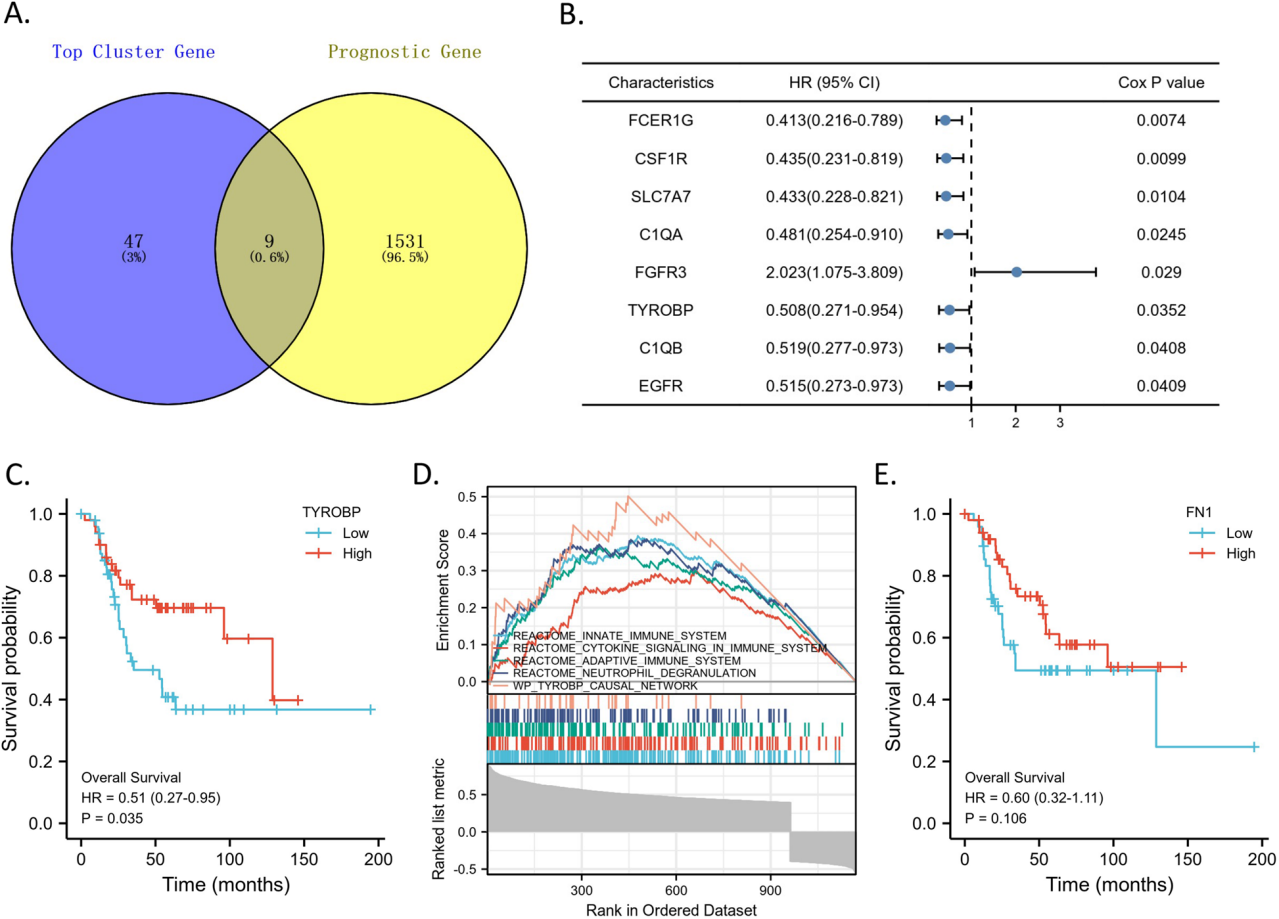
The prognostic genes in the top 5 clusters. **A** The Venn diagram representing the 9 overlapping genes between 56 of top 5 cluster genes and all prognostic genes identified by univariate Cox regression analysis on TARGET-OS dataset. **B** The forest plot showing the Hazard Ratio with Credible Interval and P value of each gene in the analysis. **C** Survival curve showing OS overall survival based on the median expressional level cutoff of *TYROBP*. **D** GSEA plot showing significant enrichment of immune-related Reactome pathways in *TYROBP* positively co-expressed genes. **E** Survival curve showing OS overall survival based on the median expressional level cutoff of *FN1*

To furtherly explore the potential effect of the 5 top clusters on OS patient prognosis, we did univariate Cox regression based on TARGET-OS database and found 9 of the top cluster genes can also predict the prognosis of OS patients (Fig. 3A-B). Interestingly, 5 of the 9 prognostic genes, i.e., *FCER1G*, *CSF1R*, *C1QA*, *TYROBP*, and *C1QB*, were from the TYROBP-centered top 1 cluster, suggesting the importance of top 1 cluster in OS progression (Fig. 3B). All the 5 genes from top 1 cluster, including *TYROBP* itself (Fig. 3C), had a Hazard Ratio (HR) less than 1 (Fig. 3B), suggesting their protective effect on good OS prognosis (i.e., the higher expression associated with good prognosis). By GSEA enrichment analysis, the *TYROBP* co-expressed genes (|*R*|> 0. and *p* < 0.05) in OS were enriched in annotations including innate/adaptive immune system, cytokine signaling, neutrophil degranulation, and TYROBP causal network (Fig. 3D), indicating the strong connection between *TYROBP* and immune functions. Moreover, although FN1 had the highest degree in the whole network, it did not significantly impact the OS prognosis (Fig. 3E).

### The conserved *TYROBP* co-expression network across multiple cancer types and its classic immune activation function

**Fig. 4:**
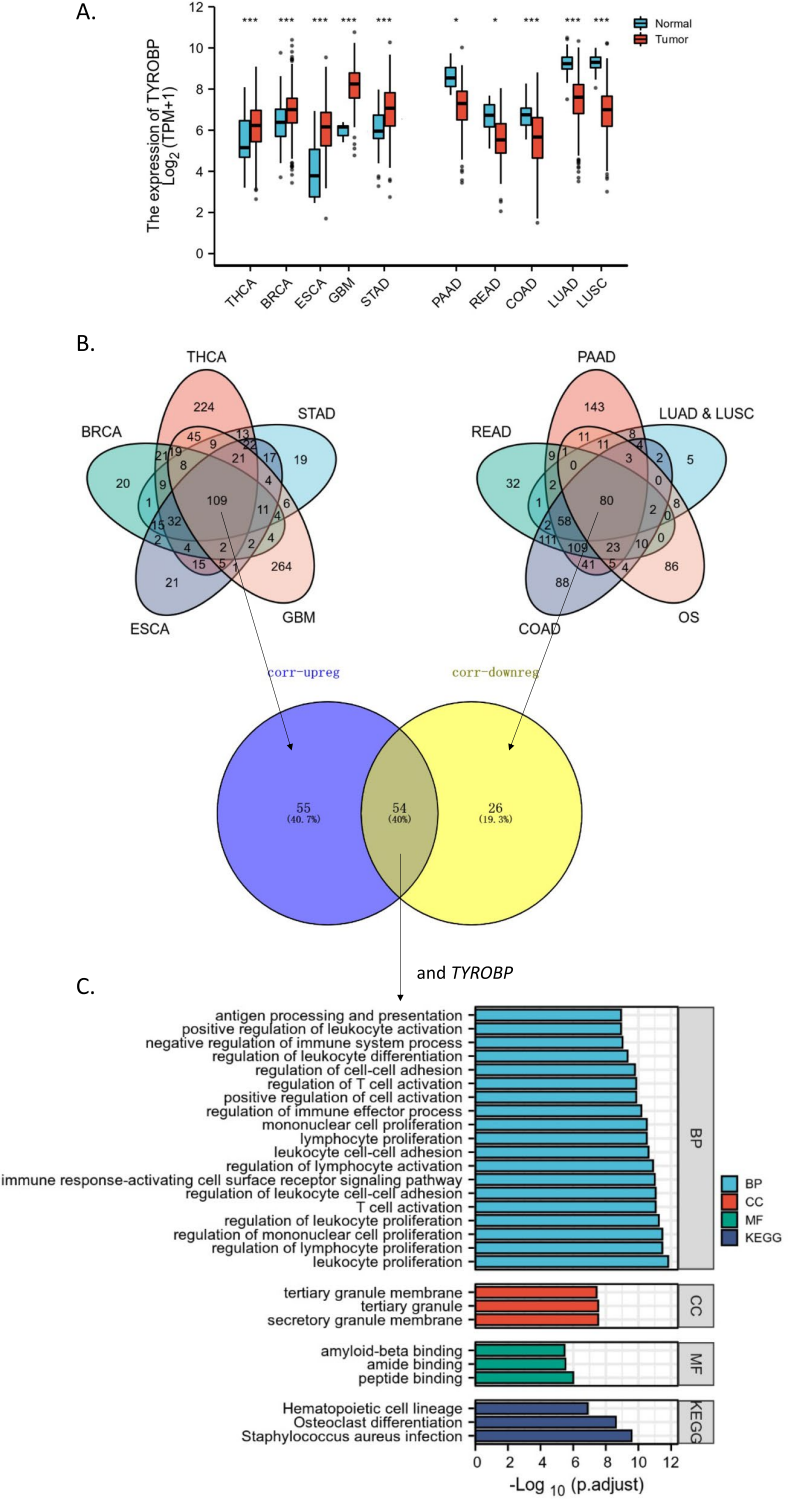
The common 55 *TYROBP* co-expression genes (including *TYROBP*) across cancer types and their enriched functions. **A** The boxplot showing the expressional levels of TYROBP between para-cancer tissues and cancer tissues across different cancer types (*, *p* < 0.05; **, *p* < 0.01; ***, *p* < 0.001). **B** The Venn diagram representing the number of *TYROBP* co-expression genes in cancer types with upregulated *TYROBP* (Left) and cancer type with downregulated *TYROBP* (Right), and the 55 of common genes across all cancer types. **C** The top 20 in all annotations and the top 3 in each type of database (BP, CC, MF, KEGG) enriched by the 55 *TYROBP* co-expression genes

As the second highest degree gene in DEGs network and the highest degree gene in top 1 cluster, *TYROBP* also significantly impact the prognosis of OS patients (Fig. 3C). Therefore, we furtherly identified the conserved *TYROBP* co-expressed network across multiple cancer types to explore its classic function in tumor progression. Firstly, we found that the expressional level of *TYROBP* was significantly (adjusted *p* < 0.05) lower in cancers including READ, PAAD, LUAD, LUSC, COAD, and OS, while the expressional level was higher in cancers including THCA, BRCA, STAD, ESCA, and GBM (Fig. 4A). Then we integrated all the down-regulated co-expression genes of *TYROBP* in READ, PAAD, LUAD, LUSC, COAD, and OS, and all the up-regulated co-expression genes in THCA, BRCA, STAD, ESCA, and GBM (Fig. 4B). By overlapping the 80 of down-regulated co-expression genes and 109 up-regulated co-expression genes, we got 54 of conversed *TYROBP* co-expressed genes (*R* > 0.6 and *p* < 0.05) across multiple cancer types (Fig. 4B). These genes were enriched in functions such as mononuclear cell/lymphocyte/leukocyte proliferation, T cell activation, and antigen processing and presentation (Fig. 4C), suggesting a classic immune activation function and a potential immune infiltration-associated function.

### A two-gene signature (FCER1G and SPI1) from *TYROBP* co-expression network can predict OS prognosis

**Fig. 5:**
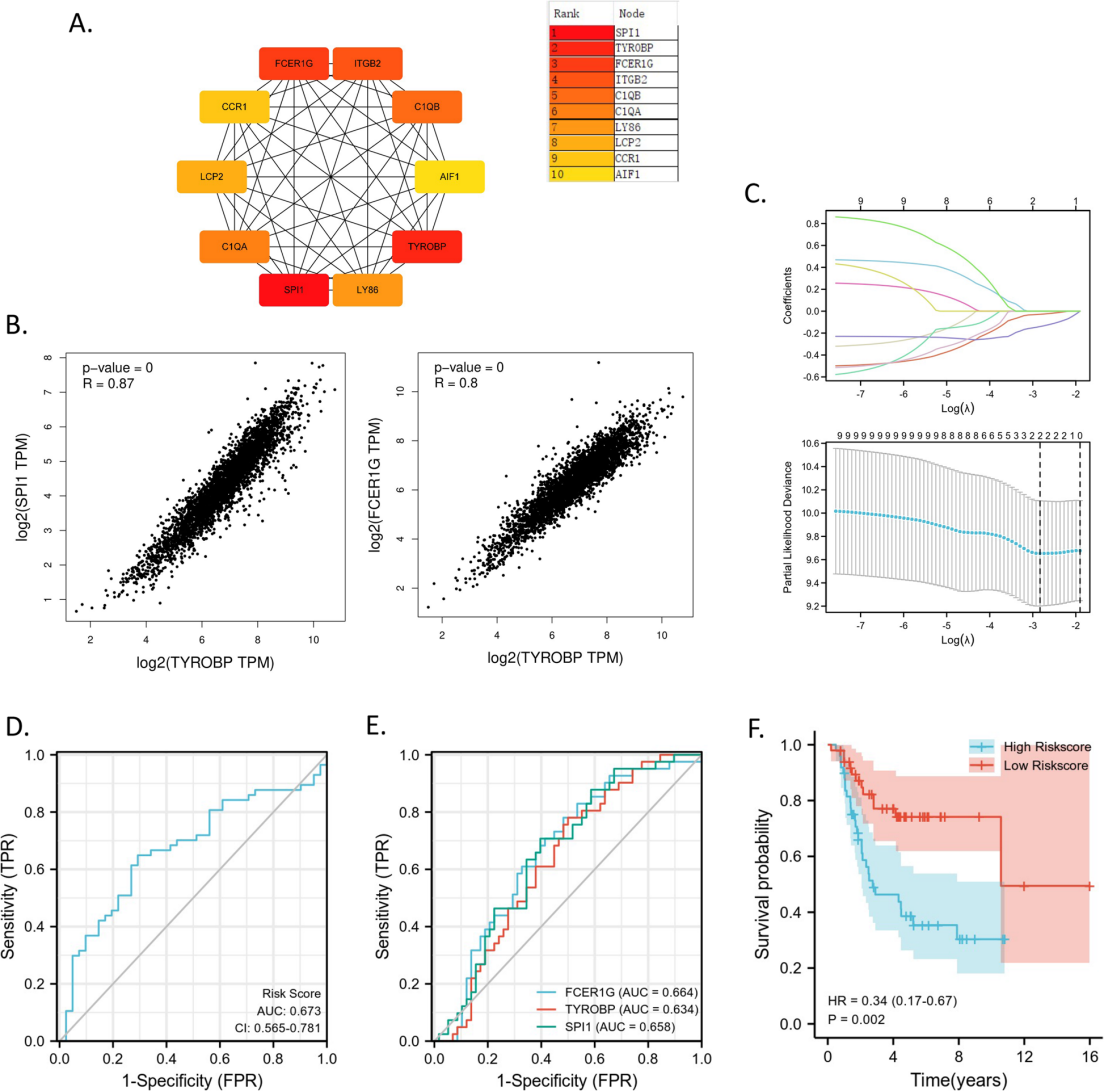
The prognostic two-gene signature from the hub genes in conserved SPI1-TYROBP-FCER1G network. **A** The hub genes in conserved SPI1-TYROBP-FCER1G network. The depth of color reflecting the rank in the hub genes. **B** The correlation between *SPI1*/*FCER1G* and *TYROBP* across cancer types. **C** Coefficients of selected features are shown by lambda parameter. Partial likelihood deviance versus log (λ) was performed through LASSO regression. **D** ROC curve based on the two-gene signature for overall survival according to risk scores. **E** ROC curves for overall survival according to the expressional levels of *SPI1*, *FCER1G*, or *TYROBP*. **F** Kaplan–Meier plots of overall survival according to risk scores

To explore the effect of the conserved TYROBP co-expression network (Supplementary Fig. 1C) on OS prognosis, we identified the top 10 hub genes in the network by PPI analysis and defined a two-gene signature from the 10 hub genes by the overall survival-based LASSO Cox regression model. Firstly, we found the 10 hub genes including *SPI1* (first rank), *TYROBP* (second rank), *FCER1G* (third rank), *ITGB2*, *C1QB*, *C1QA*, *LY86*, *LCP2*, *CCR1*, and *AIF1* (Fig. 5A). Even across the ten cancer types in Fig. 4B, the expressional levels of *SPI1*, *TYROBP*, *FCER1G* were also highly correlated with *R* > 0.8 and *p* = 0 (Fig. 5B). Therefore, we also name the *TYROBP* co-expression network as SPI1-TYROBP-FCER1G network. Furthermore, a LASSO Cox regression analysis identified an optimal two-gene predictive signature (Fig. 5C) with risk score as follows: (-0.1405) × *FCER1G* expression value + (-0.0282) × *SPI1* expression value. The AUC of risk score was 0.673 as independent prognostic indicator (Fig. 5D), which was higher than the AUCs (Fig. 5E) of *FCER1G* (0.664), *TYROBP* (0.634), and *SPI1* (0.658) expression value respectively. The overall survival of OS patients with higher risk score is significantly shorter than those with lower risk score (cut off by median risk score) from overall survival curve (Fig. 5F).

### Different levels of immune infiltration between the two clusters of OS patients based on the expressional levels of *FCER1G* and *SPI1*

**Fig. 6:**
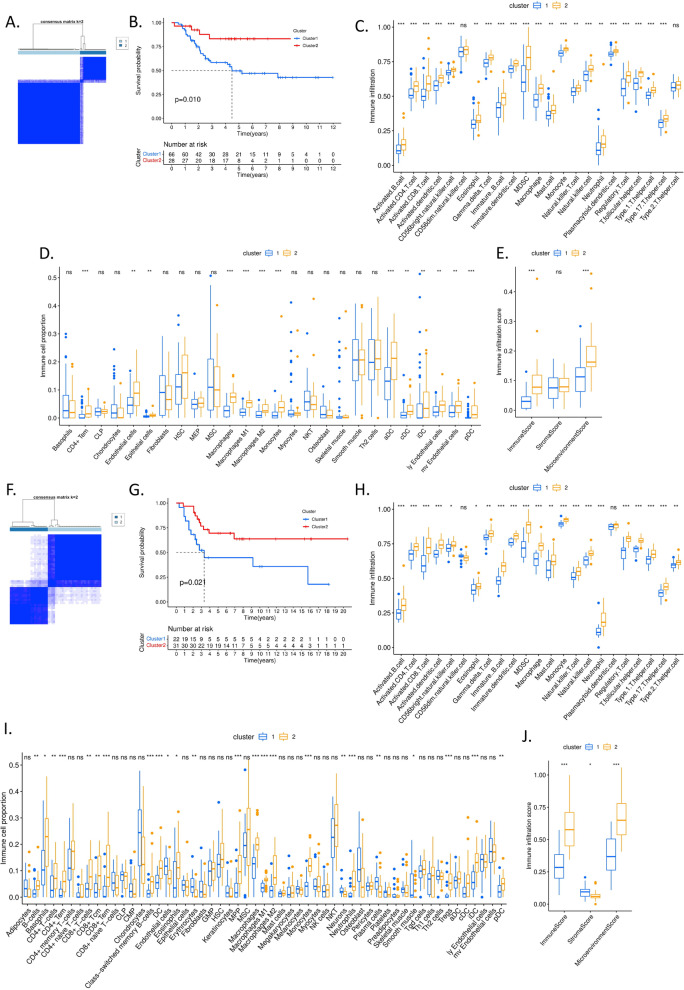
Differential overall survival and immune infiltration of OS in cluster 1 and cluster 2 subgroups according to the expressional levels of *SPI1* and *FCER1G*. **A-E** Based on TARGET-OS dataset. **F-J** Based on GSE21257 dataset. **A** and **F** Consensus clustering matrix for k = 2. **B** and **G** Kaplan–Meier overall survival curves for OS. **C** and **H** The boxplot of immune cell infiltration score in cluster 1 and 2. **D** and **I** The boxplot of immune cell infiltration proportion in cluster 1 and 2. **E** and **J** The boxplot of immune infiltration score in cluster 1 and 2. ns, *p* ≥ 0.05,*, *p* < 0.05,**, *p* < 0.01,***, *p* < 0.001

Because dividing OS patients by median risk score is relative arbitrary, we divided OS patients according to the expressional levels of *FCER1G* and *SPI1* in TARGET-OS database by the consensus clustering analysis. We found cluster 1, which had lower expressional levels of *FCER1G* and *SPI1*, was significantly associated with poorer overall survival (*p* = 0.010, Fig. 6A). In addition, cluster 1 also had lower stromal and immune scores (*p* < 0.01 and *p* < 0,001 separately, Fig. 6B), compared with cluster 2. To explore the alteration of different immune cell subsets, we estimate the immune cell infiltrations via both ssGSEA algorithm and xCell platform. We found almost all immune cells had higher scores in cluster 2 than that in cluster 1 in addition to CD56^dim^ NK cells and Th2 cells (Fig. 6C). Besides, there were higher proportions of CD4 + Tem cells, endothelial cells (including ly and mv endothelial cells), macrophages (including M1 and M2), monocytes, dendritic cells (including aDC, cDC, iDC and pDC) in cluster 2 than that in cluster 1 (Fig. 6D), after filtering cell subset less than 1% (0.01). Furthermore, xCell also identified the significantly higher immune score and microenvironment score in cluster 2 than that in cluster 1, suggesting more immune infiltration and lower tumor microenvironment abundance (Fig. 6E).

To validate the result, we did the same analyses on GSE21257 data. Similarly, cluster 1 had significantly poorer overall survival compared with cluster 2 (*p* = 0.021, Fig. 6F-G). Almost all immune cells had higher scores in cluster 2 than that in cluster 1 in addition to CD56^dim^ NK cells and pDC cells (Fig. 6H). Besides, there were higher proportions of CD4^+^ T cells (including T cells and Tem), CD8^+^ T cells (including T cells, Tcm and Tem), B cells (including B cells and class-switched memory B cells), endothelial cells, macrophages (including M1 and M2), monocytes, dendritic cells (including iDC and pDC), basophils, eosinophils, erythrocytes, DC, and iDC in cluster 2 than that in cluster 1 (Fig. 6I), after filtering cell subset less than 1%. However, there were lower proportions of plasma cells and smooth muscle cells in cluster 2 than cluster 1 (Fig. 6I). Similarly, xCell also identified the significantly higher immune score/microenvironment score but lower stroma score in cluster 2 than that in cluster 1, indicating higher immune infiltration but lower tumor purity and stroma cell proportion (Fig. 6J).

These results indicated that low expressional levels of *FCER1G* and *SPI1* could predict an attenuated immune infiltration and poorer prognosis, suggesting a potential immune escape and metastasis tendency. By comparing the expressional level between OS patients with metastases after diagnosis of primary tumor and patients without metastases in GSE21257, we found the expressional levels of *TYROBP* (*p* = 0.0003, Log_2_FC = -1.16), *FCER1R* (*p* = 0.0002, Log_2_FC = -1.19) and *SPI1* (*p* = 0.0059, Log_2_FC = -0.599) were all down-regulated in metastases group, consistent with the above result.

### Transcription factor SPI1 may initiate the expression of whole TYROBP network and be impacted by upstream TNF-α

**Fig. 7:**
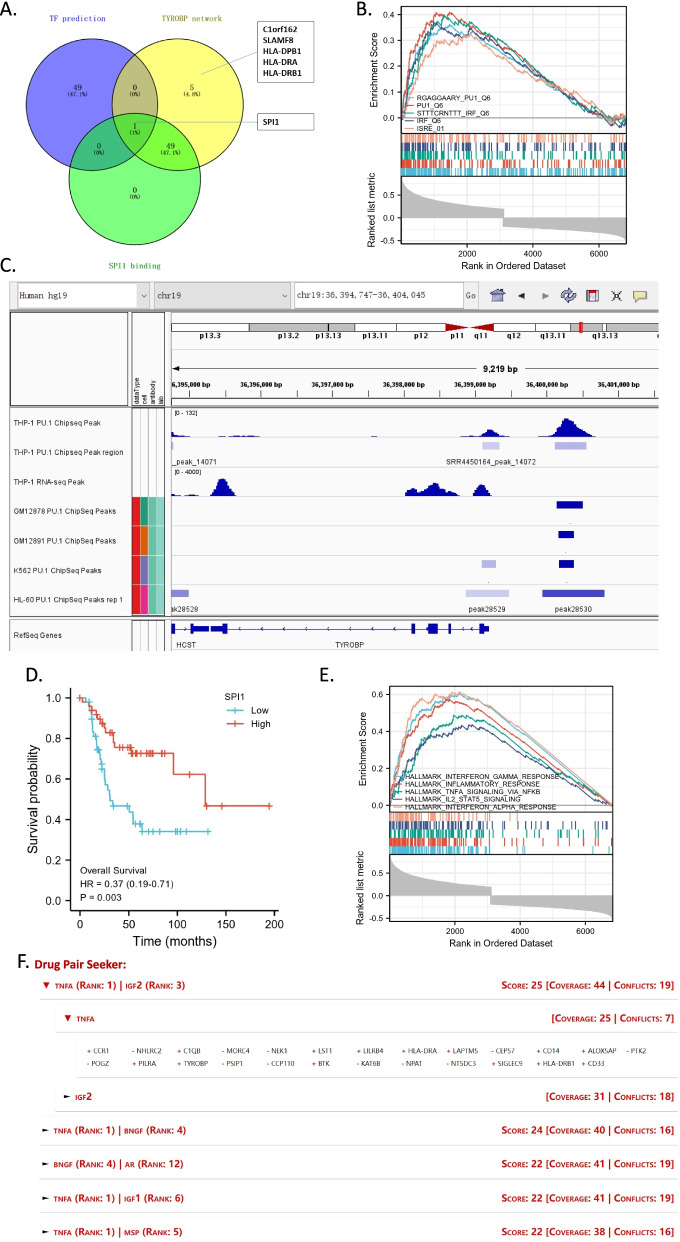
The key transcription factor SPI1 in the conserved SPI1-TYROBP-FCER1G network. **A** The Venn diagram representing the numbers of ChEA3 predicted transcription factors/SPI1 binding genes and TYROBP network genes. **B** GSEA plot showing significant enrichment of PU.1 and IRF binding motifs in *TYROBP* positively co-expressed genes. **C** The genomic signal density around *TYROBP* showing the PU.1 binding regions upstream and the mRNA level of *TYROBP*. **D** Kaplan–Meier plots of overall survival according to expressional level of SPI1 (median cutoff). **E** GSEA plot showing the significant enrichments of signaling pathways including TNF-α signaling via NF-κB in *TYROBP* positively co-expressed genes. **F** Drug pairs found by Drug Pair Seeker representing the cytokine pairs including TNF-α that can upregulate the TYROPB network

Although TYROBP is in the center of its whole co-expression network, it cannot regulate gene expression as a transcription factor. Therefore, we predicted the key transcription factor of the 55 genes of the TYROBP network by ChEA3 platform. SPI1 ranked second in the top 50 predicted transcription factors. Furthermore, in addition to the 5 genes (i.e., C1orf162, SLAMF8, HLA-DPB1, HLA-DRA, and HLA-DRB1), all other 50 genes in the network, including *TYROBP*, *FCER1G*, and *SPI1* itself, can be bound and regulated by SPI1 (Fig. 7A). Interestingly, GSEA enrichment analysis also showed that transcription factor PU.1/SPI1 motifs were enriched in *TYROBP* positively correlated genes (Fig. 7B). ChIP-seq and RNA-seq data validated the binding of SPI1 on *TYROBP* (Fig. 7C) and other 9 of network hub genes (data not shown). Notably, the low expressional level of *SPI1* was significantly associated with poorer overall survival in TARGET-OS dataset (Fig. 7D).

To identifying the upstream factor of SPI1 in OS, by GSEA analysis, we found ‘TNF-α signaling via NF-κB’, ‘IFN-α/γ response’, ‘inflammatory response’ and ‘IL2-STAT5 signaling’ pathways were all enriched in *TYROBP* positively correlated genes (Fig. 7E). Moreover, by the analysis of Drug Pair Seeker (DPS), we found TNF-α and IGF2 can partly rescue the under-expression of *TYROBP* network and the over-expression of *TYROBP* negatively-correlated genes in OS (Fig. 7F). These results suggested the importance of TNF-α in the activation of SPI1 and TYROBP network, and a potential deficiency of tumor microenvironmental TNF-α in OS and OS with poorer prognosis.

### The TYROBP network-associated immune molecules affecting immune infiltration and activation

**Fig. 8:**
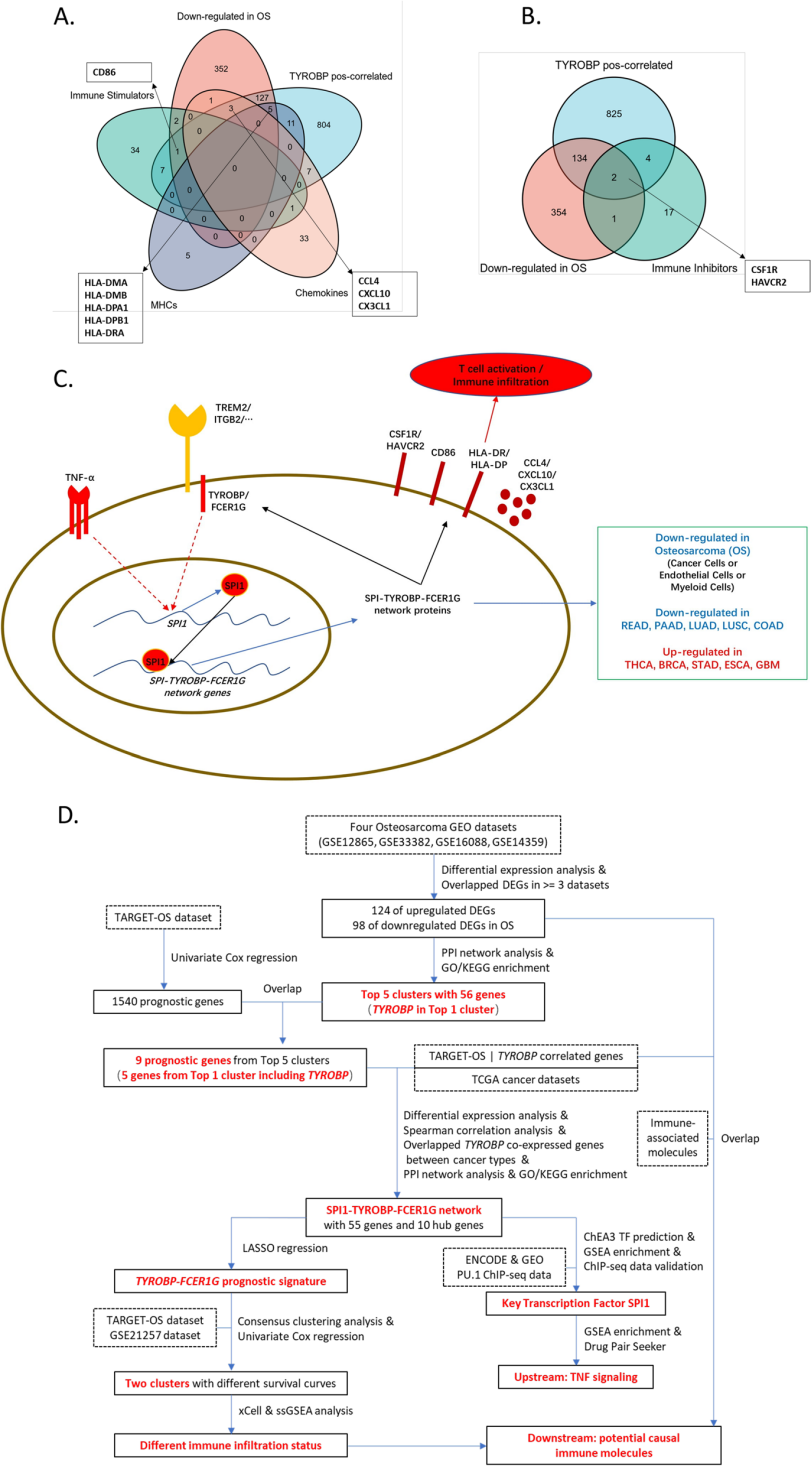
The relationship between TYROBP network and immune-related genes in OS and the schematic workflow of the whole study. **A-B** The Venn diagram representing the overlapping between downregulated genes in OS, TYROBP positively correlated genes in OS, and immune related genes (i.e., immune stimulators, MHC molecules, chemokines in **A**, and immune inhibitors in **B**). **C** The working model representing the TNF-α-SPI1-TYROBP network pathway across cancer types, which can regulate immune infiltration and CD8^+^ T cell activation. **D** Workflow for the whole study as to how to identify the SPI1-TYROBP-FCER1G network and its association with immune microenvironment

To furtherly explore the mechanism of downregulated immune infiltrations in OS, we overlapped the immune stimulators, MHC molecules, chemokines, and immune inhibitors with 965 of TYROBP positively-correlated genes (*R* > 0.4, *p* < 0.05) in TARGET-OS dataset and 491 of down-regulated genes in >  = 2 GEO datasets (i.e., GSE33382, GSE12865, GSE16088 and GSE14359). We identified 1 of immune stimulators (CD86), 5 of MHC molecules (HLA-DMA, HLA-DMB, HLA-DPA1, HLA-DPB1, HLA-DRA), 3 of chemokines (CCL4, CXCL10, CX3CL1), and 2 of immune inhibitors (CSF1R, HAVCR2), which were both TYROBP positively-correlated and down-regulated in OS samples compared with that in normal samples (Fig. 8A-B), which potentially underlay the mechanism of attenuated immune infiltrations by the under-expression of SPI1-TYROBP-FCER1G network in OS (Fig. 8C).

## Discussion

This study identified a conserved *TYROBP* co-expression network that was associated with both OS oncogenesis and prognosis. The whole workflow was summarized in Fig. 8D. Firstly, we found five functional gene clusters of DEGs between control and OS samples. Then we focused on the TYROBP-centered top 1 cluster that was down-regulated in OS and associated with MHC II complex, immune cell activation and cytokine production. The under-expression of five genes (i.e., *FCER1G, CSFR1, C1QA, TYROBP*, and *C1QB*) from top 1 cluster were also associated with poorer overall survival of OS patients. Furthermore, we extended the top 1 cluster into a more conserved *TYROBP* co-expression network with 10 hub genes (*SPI1, TYROBP, FCER1G, ITGB2, C1QB, C1QA, LY86, LCP2, CCR1, AIF1*) by an analysis across cancer types, which was associated with immune cell proliferation and T cell activation. Then, the risk score combining the expression levels of both *SPI1* and *FCER1G* were constructed to predict the overall survival of OS patients. Moreover, we found the distinguished immune infiltrations between two clusters of OS patients with different overall survival times, suggesting the importance of SPI1-TYROBP-FCER1G network in upregulating the microenvironmental immune infiltration and promoting better prognosis. In addition, we identified SPI1 as the key transcription factor in the network, and found it’s potentially associated with the upstream TNF-α signaling. We also identified the TYROBP positively-correlated and downregulated immune molecules in OS, suggesting the immune-related mechanism of attenuated immune infiltration and activation in OS.

As a conserved and immune-related network across multiple cancer types, some molecules in the SPI1-TYROBP(i.e., KARAP or DAP12)-FCER1G network have been identified as prognostic biomarkers before in other cancer types [38–41] and OS [42–44]. Although the network was upregulated in certain cancer types (e.g., clear cell renal cell carcinoma, breast cancer, gastric cancer, and low-grade glioma) and positively associated with the poorer prognosis and higher immune infiltrations, the network was downregulated in OS, positively associated with higher immune infiltrations in OS, and negatively associated with the poorer prognosis of OS. Therefore, despite the same positive-correlation between TYROBP network and higher immune infiltrations across multiple cancer types [38–45], the associations between TYROBP network and overall survival in different cancer types were different, suggesting the altered role of immune infiltration or altered major infiltrating cell types among different cancer types. In a word, SPI1-TYROBP-FCER1G network is a conserved immune-related network underlying both the oncogenesis and the prognosis of OS and other cancer types.

Across multiple cancer studies, the TYROBP network was always positively-associated with immune infiltration, such as the increased infiltration of CD8^+^ T cells, DCs, and macrophages [38–45]. According to our study on its potential mechanism, the overexpression of *TYROBP* network may activate the immune infiltration by the co-expressed immune stimulators, MHC molecules, and chemokines. After recruiting immune cells into the tumor microenvironment by secreting CCL4 (collaborating with CCR5 to recruit CD8 + T cells, NK cells, monocytes, etc. [46, 47]), CXCL10 (collaborating with CXCR3 to recruit CD8 + T cells, NK cells, etc. [48–50]), and CX3CL1 (not only collaborating with CX3CR1 to recruit CD4 + T cells, CD8 + T cells, dendritic cells [51, 52], but also inducing OS metastasis via ICAM-1 [52]), molecules CD86 (co-stimulatory molecule CD86 polymorphism is also associated with increased susceptibility to osteosarcoma [53]) and MHCs (HLA-DPA1/HLA-DPB1/HLA-DRA) on the surfaces of the tumor cells, endothelial cells or DCs/macrophages can furtherly activated T cells in the microenvironment. Therefore, the under-expression of TYROBP network can lead to the attenuated immune infiltrations of OS due to the lack of chemokines, stimulators and MHCs, which can cause the immune escape and then the metastasis of OS. Furthermore, we also found the down-regulation of immune checkpoint inhibitors CSF1R and HAVCR2 in OS. It has been found that OS tumor cell-intrinsic CSF1R can enhances cell proliferation and metastasis by CSF-1R/JAG1 axis or ERK signaling pathway [54, 55]. HAVCR2 (i.e., TIM-3), as an immune checkpoint inhibitor, can also promote the OS progression by inducing T cell exhaustion and impair T cell function when expressed on T cells [56, 57] and inducing M2 macrophage polarization when expressed on macrophages [58, 59]. The relationship between the under-expression of CSF1R and immune infiltration in OS needs further study in the future.

As transmembrane immune signaling adaptor proteins, TYROBP and FCER1G may mediate an intracellular signal via immunoreceptor tyrosine-based activation motif (ITAM) to up-regulate the expression of *SPI1* and then initiate the whole SPI1-TYROBP-FCER1G network for a competent immunological surveillance function with upregulated immune infiltrations in tumor microenvironment [60–63]. SPI1, as an oncogene, is also an important transcription factor for monocyte and macrophage identity. However, it can also be regulated by multiple signals, such as NF-κB signaling from upstream TYROBP, FCER1G [62, 63], TNF-α [64, 65] or IFN-γ [66, 67]. SPI1, as the key transcription factor for the network, may shape a positive-feedback network with the signaling from TYROBP/FCER1G or environmental TNF-α/IFN-γ, then furtherly initiate the whole SPI1-TYROBP-FCER1G network to regulate the immune infiltration and cancer prognosis (Fig. 8C). In OS, the under-expression of SPI1-TYROBP-FCER1G network in tumor cells or microenvironmental endothelial cells/myeloid cells can potentially attenuate the immune infiltration and T cell activation, leading to the metastasis/poorer prognosis of OS (Fig. 8C).

This study has several limitations. First, the relatively small number of DEGs that appeared in >  = 3 datasets may be due to the heterogeneity between these GEO datasets that we have mentioned in the result, which can reduce the efficiency of finding more functional gene clusters. However, it did not affect the validity of already identified results. In addition, although current studies have obtained useful findings, more laboratory studies both in vitro and in vivo are needed to validate these bioinformatic results in the future, such as the levels of SPI1-TYROBP-FCER1G network-regulated immune molecules in OS tumor cells. Thirdly, in addition to OS tumor cells, the expressional levels of SPI1-TYROBP-FCER1G network in other potential cell types, such as myeloid cells and endothelial cells, in the microenvironment are still unclear. Furthermore, the reason why SPI1-TYROBP-FCER1G network was upregulated in certain cancer types but downregulated in the others including OS, and its potential immunological mechanism, cannot be answered by our study and need further explorations in the future.

In a word, our study identified a conserved SPI1-TYROBP-FCER1G network across cancer types for the first time, and explored its immune-related mechanism in OS, which could be used as a treatment target in OS with TNF-α or TYROBP/FCER1G stimulators to rescue the under-expression of SPI1-TYROBP-FCER1G network in OS. The drug seeker analysis also supported a standpoint [68] that anti-TNF-α treatment in OS may cause metastasis and poorer prognosis by sustaining the under-expression of SPI1-TYROBP-FCER1G network. We provided an essential bioinformatic mechanism foundation and a novel therapeutic target of OS oncogenesis and prognosis for future explorations.

## Supplementary Information

**Additional file 1: Figure 1.** Differentially expressed genes, DEGs network, and SPI1-TYROBP-FCER1G network. **A**. The volcanos of DEGs in four OS datasets. The X-axis represented the value of log2 (fold change), while the Y-axis represented the value of -log10 (adjusted *p* value); the red node represented upregulated genes, while the blue node represented the downregulated genes. **B**. The PPI network of DEGs. The area represented the degree of each node; the red node represented upregulated genes, while the blue node represented the downregulated genes. **C**. The SPI1-TYROBP-FCER1G network. The area represented the degree of each node; the depth of color reflecting the rank in the hub genes.

## Data Availability

Publicly available datasets were analyzed in this study. The data can be found here: https://www.ncbi.nlm.nih.gov/geo/query/acc.cgi (GEO datasets), https://xenabrowser.net/datapages/ (TCGA datasets), and https://ocg.cancer.gov/programs/target/data-matrix (TARGET-OS dataset).

## Cell Type

aDC: Activated dendritic cells
cDC: Conventional dendritic cells
iDC: Immature dendritic cells
pDC: Plasmacytoid dendritic cells
Tcm: Central memory T-cells
Tem: Effector memory T cells
Tgd: T cells gamma delta
CMP: Common myeloid progenitors
GMP: Granulocyte–macrophage progenitors
MSC: Mesenchymal stem cells
CLP: Common lymphoid progenitors
ly endothelial: Lymphatic endothelial cells
mv endothelial: Microvascular endothelial cells

## Cancer Type

OS: Osteosarcoma
THCA: Thyroid carcinoma
BRCA: Breast invasive carcinoma
ESCA: Esophageal carcinoma
GBM: Glioblastoma multiforme
STAD: Stomach adenocarcinoma
PAAD: Pancreatic adenocarcinoma
READ: Rectum adenocarcinoma
COAD: Colon adenocarcinoma
LUAD: Lung adenocarcinoma
LUSC: Lung squamous cell carcinoma
