# Supplementary figures and images for: The role of SPI1-TYROBP-FCER1G network in oncogenesis and prognosis of osteosarcoma, and its association with immune infiltration
Source: BMC Cancer. 2022 Jan 25;22:108. doi: 10.1186/s12885-022-09216-w (PMC8790913; doi:10.1186/s12885-022-09216-w)

Supplementary Figure 1.

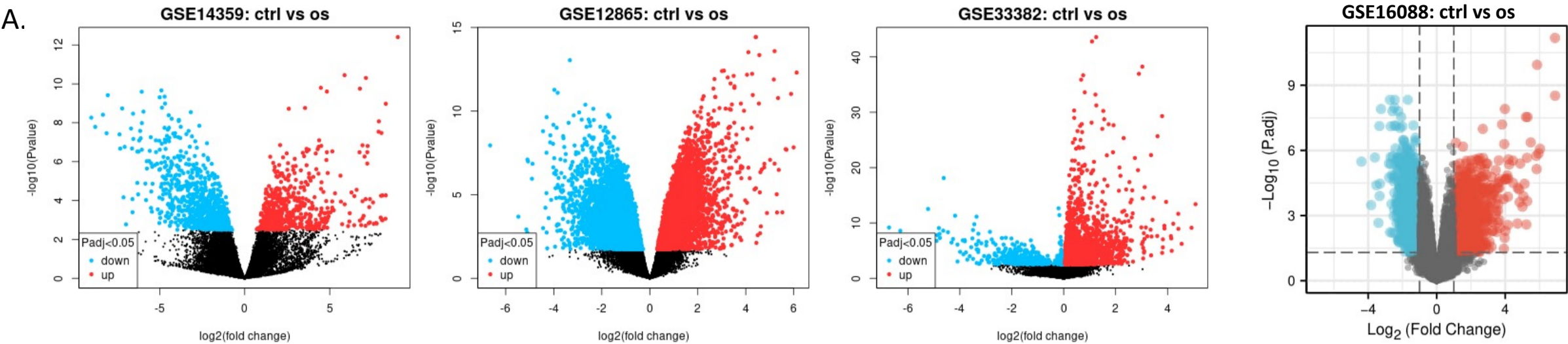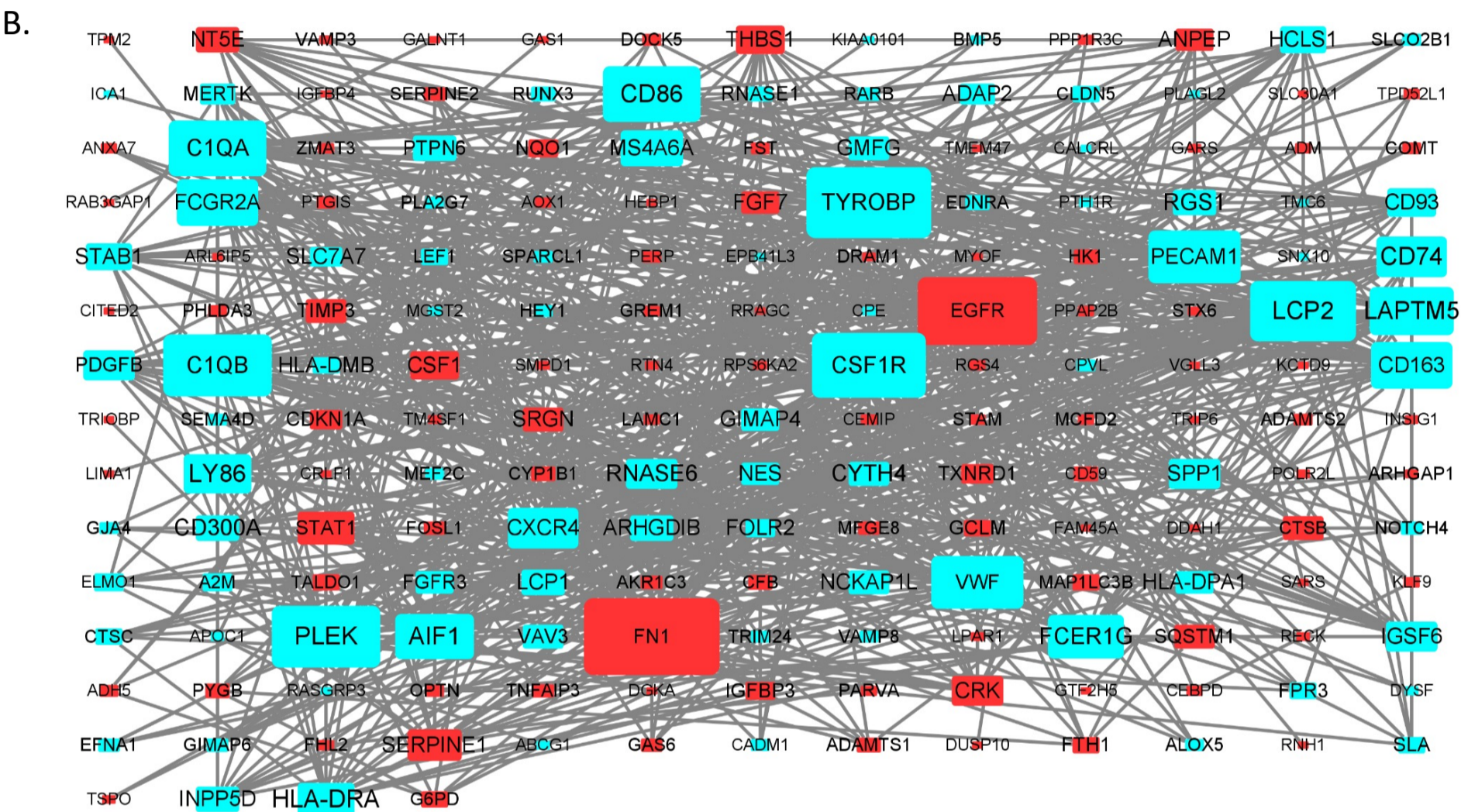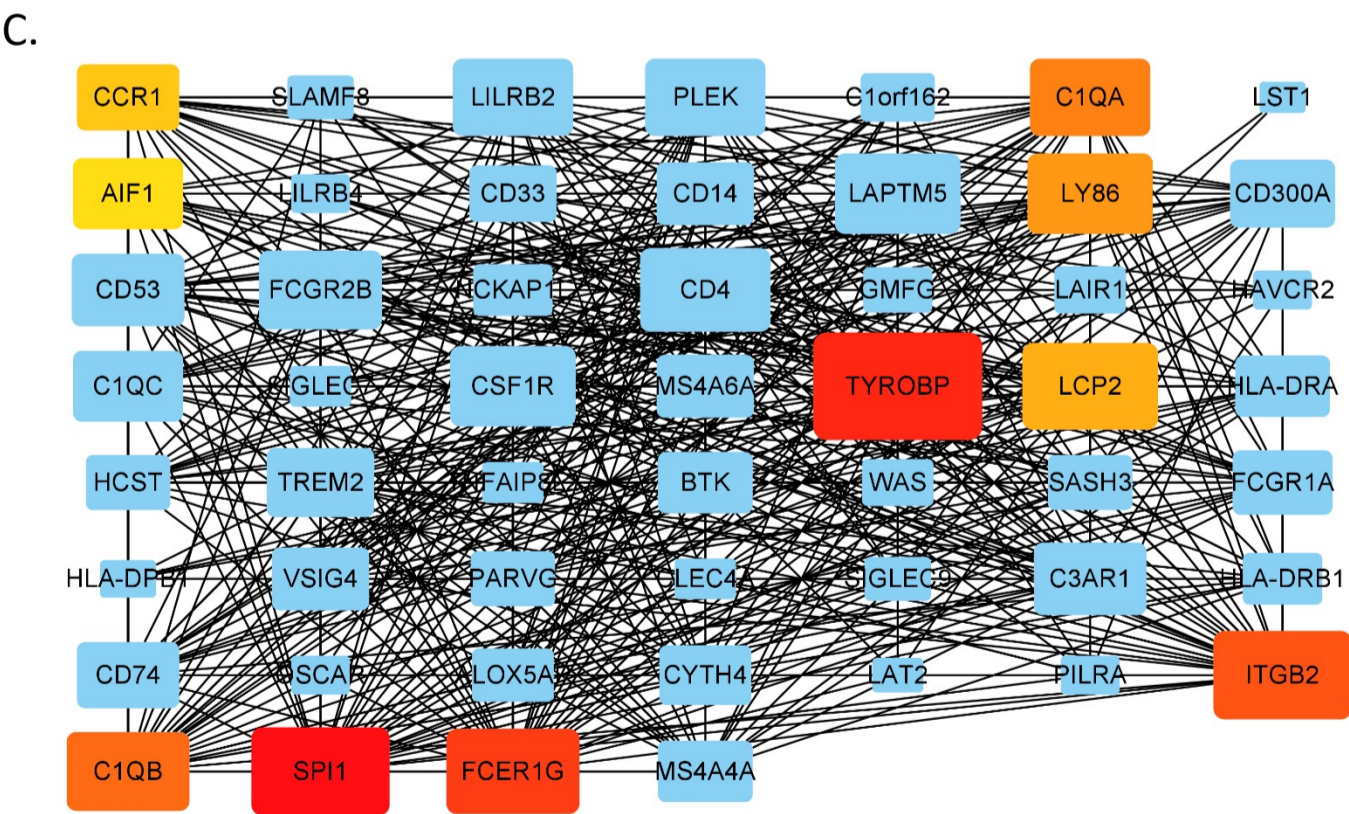

Supplement: Supplementary file 1 — Additional file 1: Figure 1. Differentially expressed genes, DEGs network, and SPI1-TYROBP-FCER1G network. A. The volcanos of DEGs in four OS datasets. The X-axis represented the value of log2 (fold change), while the Y-axis represented the value of -log10 (adjusted p value); the red node represented upregulated genes, while the blue node represented the downregulated genes. B. The PPI network of DEGs. The area represented the degree of each node; the red node represented upregulated genes, while the blue node represented the downregulated genes. C. The SPI1-TYROBP-FCER1G network. The area represented the degree of each node; the depth of color reflecting the rank in the hub genes. [file 12885_2022_9216_MOESM1_ESM.pdf]
